# Unravelling the molecular network of desiccation tolerance in resurrection plants started with the model plant *Craterostigma plantagineum*

**DOI:** 10.1007/s00425-025-04752-8

**Published:** 2025-06-25

**Authors:** Dorothea Bartels, Valentino Giarola, John Chandler

**Affiliations:** 1 Institute of Molecular Physiology and Biotechnology of Plants (IMBIO), Faculty of Natural Sciences, University of Bonn, Kirschallee 1, 53115 Bonn, Germany; 2 Hudson River Biotechnology, Nieuwe Kanaal, 7 V 6709PA Wageningen, The Netherlands; 3 Department of Plant Developmental Biology, Max Planck Institute for Plant Breeding Research, Carl-Von-Linné-Weg, 50829 Cologne, Germany

**Keywords:** Abscisic acid, Evolution of desiccation, LEA proteins, Seed desiccation tolerance, Vegetative desiccation tolerance

## Abstract

**Main conclusion:**

Molecular studies of desiccation-tolerant resurrection plants identified major components for surviving severe water depletion of vegetative tissues. The research also highlights potential applications for crop protection during drought.

**Abstract:**

The ability of vegetative plant tissues to withstand desiccation is a property of a small group of resurrection plants specific to specialized ecological niches. In the 1980s, studies on these plants were limited to the physiological and morphological levels. However, in 1990, a study by Bartels et al. using the South African resurrection plant *Craterostigma plantagineum* was the first to address desiccation tolerance at the molecular level. A differential screening approach with *C. plantagineum* leaves and callus pretreated with ABA led to the identification of transcripts that were upregulated by desiccation. Many of the identified genes encoded late embryogenesis-abundant (LEA) proteins, which are abundant proteins that accumulate during normal seed development. Therefore, the study confirmed that the acquisition of desiccation tolerance in vegetative tissues of resurrection plants partially involves the seed maturation programme involving ABA. Subsequent research with *C. plantagineum* contributed to elucidating the gene regulatory networks and metabolic changes that contribute to desiccation tolerance and provided the basis for studies with other resurrection species. More recently, the genomes of *C. plantagineum* and several other resurrection plants have been sequenced, which has allowed comparative genomics approaches to identify conserved mechanisms and signatures associated with vegetative desiccation tolerance. A primary goal remains to transfer existing knowledge from resurrection plants to genetically engineer drought tolerance in crop plants, which will improve survival during periods of drought and will maintain future food security despite increasing impacts of climate change.

## Desiccation-tolerant resurrection plants

**Fig. 1:**
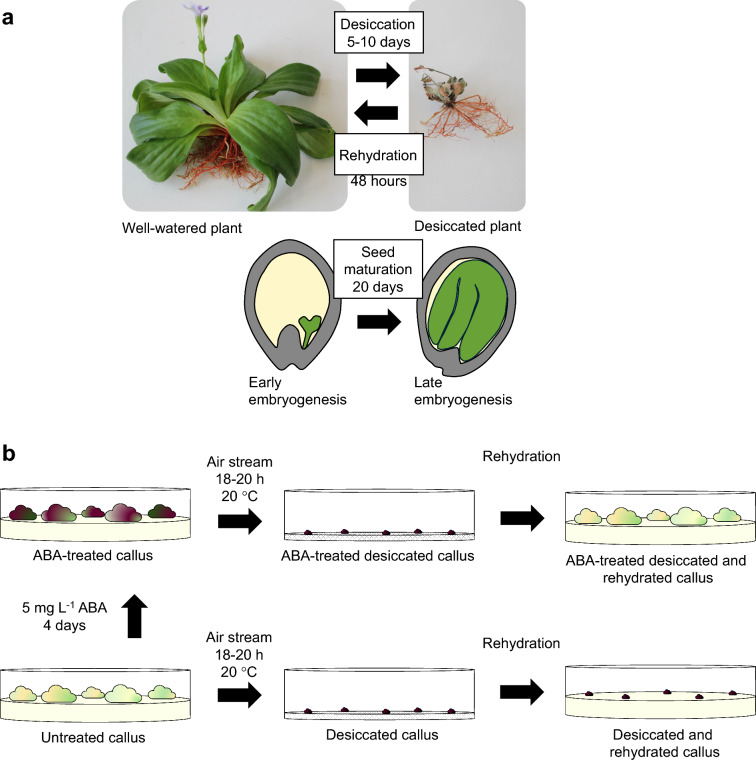
Desiccation tolerance in *Craterostigma plantagineum*. **a** Acquisition of desiccation tolerance in *Craterostigma plantagineum* plants and dicot seeds. Upper panel: In dehydration–rehydration experiments, well-watered whole plants or detached leaves are air-dried until complete desiccation, during which more than 90% of water is lost. Plants can remain desiccated for several days or even months and restore full metabolic activities within 48 h upon rewatering. Lower panel: cartoon of a dicot seed during early and late embryogenesis phases when maturation involves the acquisition of desiccation tolerance. **b** Study of desiccation tolerance in *Craterostigma plantagineum* callus. Callus of *C. plantagineum* is sensitive to rapid desiccation, and cells cannot restore their metabolic activities upon dehydration and rehydration. However, treatment with the hormone ABA (5 mg L^-1^) for 4 days induces desiccation tolerance. Only callus pretreated with ABA can rehydrate and proliferate when transferred to plates with nutrient medium

Plant physiology and development is strictly based on an appropriate aqueous cellular environment within plant tissues, and loss of cellular water leads to irreversible damage. In fact, the ability to withstand transient desiccation is part of the developmental programme of most seeds and pollen, whereas vegetative structures start to wilt and eventually die, when the cellular water content falls below a certain threshold (Zhang and Bartels 2018). However, some mosses, ferns, pteridophytes and a small group of angiosperm plants termed resurrection plants have acquired desiccation tolerance. Resurrection plants can withstand desiccation of vegetative tissues and recover upon rehydration (Gaff 1971; Alpert 2000). The evolution of desiccation tolerance was probably a prerequisite for the transition from life in water to life on land (Barthlott et al. 2022). In the 1970s, several plants were described among the South African flora that could withstand virtually complete desiccation (Gaff 1971; 1977). These plants include *Craterostigma plantagineum* Hochst (Fig. 1). Currently, several hundred species of vascular plants are known to survive desiccation of their vegetative tissues, including dicots and monocots (Porembski 2021). Resurrection plants occur in restricted ecological niches that receive occasional rainfall. These include so-called inselbergs, which are rocky outcrops that are sparsely covered with soil, often in arid or semi-arid habitats in which the effects of persistent droughts are exacerbated by substrates that are impenetrable to roots (Alcantara et al. 2015).

**Fig. 2:**
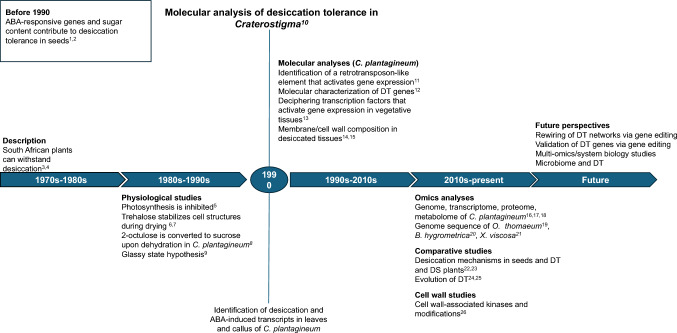
Major breakthroughs in the study of desiccation tolerance in *Craterostigma* and other resurrection plants. The study of desiccation tolerance (DT) in plants gained momentum in the early 1970s. Initial research focussed on the physiology and metabolism of resurrection plants and how metabolites that accumulate upon dehydration protect these plants from damage. The study by Bartels et al. (1990) was the first to report molecular analyses of resurrection plants, identifying genes expressed in desiccation-tolerant tissues and introducing callus as a model system. One major discovery was the identification of key regulatory components, such as CDT-1. Advancements in understanding desiccation tolerance continued when genome sequences of resurrection plants became available. These genomic data and other omics data allowed systemic and large-scale comparative analyses. Today, genomic insights combined with gene-editing technologies can now help to validate hypotheses in resurrection plants and will potentially allow the transfer of knowledge to desiccation-sensitive (DS) plants. ^1^Bartels et al. (1988); ^2^Koster and Leopold (1988); ^3^Gaff (1971); ^4^Gaff (1977); ^5^Schwab et al. (1989); ^6^Crowe et al. (1984); ^7^Schwab and Heber (1984); ^8^Bianchi et al. (1991); ^9^Leopold (1986); ^10^Bartels et al. (1990); ^11^Furini et al. (1997); ^12^Velasco et al. (1998); ^13^Bartels et al. (2007); ^14^Gasulla et al. (2013); ^15^Moore et al. (2013); ^16^Rodriguez et al. (2010); ^17^Xu et al. (2021); ^18^VanBuren et al. (2023); ^19^VanBuren et al. (2015); ^20^Xiao et al. (2015); ^21^Costa et al. (2017); ^22^VanBuren et al. (2017); ^23^Pardo et al. (2020); ^24^Giarola et al. (2018); ^25^VanBuren et al. (2018); ^26^Jung et al. (2019)

Early research on resurrection plants was mainly on the morphological and physiological levels, and the overarching goal of the research has always been the identification of protection mechanisms that enable survival during water depletion. One of the studied plants was *C. plantagineum*, and it was shown that the presence of osmolytes and sugars could protect membranes during dehydration (Schwab and Heber 1984; Schwab et al. 1989). Similarly, physiological studies in other resurrection plants focussed on osmoregulatory pathways that control the biosynthesis and transport of compatible solutes such as polyamines (Flores and Galston 1982) and osmoprotectants such as proline (Newton et al. 1986). However, it was not until the publication by Bartels et al. (1990) reported molecular analyses of *C. plantagineum* that resurrection plants attracted scientific attention. Many publications on molecular studies of different species followed, including detailed recent genomic and transcriptomic analyses (Fig. 2; Table 1). During the last 10 years, a particularly fruitful collaboration was established between the research group of D. Bartels (University of Bonn, Germany) and R. VanBuren (Michigan State University, USA), who brought large-scale transcriptomics and genome analysis to the research of resurrection plants. In addition to *C. plantagineum*, *Oropetium thomaeum* was studied as a model for grasses and a close relative of cereals (VanBuren et al. 2015). It was shown that the genome of the resurrection grass *O. thomaeum* possesses deep synteny to that of other grasses, including several cereals such as sorghum or maize.

**Table 1:** Overview of selected reviews^a^ summarizing research of the timeline depicted in Fig. 2

Title	References
Gene expression in response to abscisic acid and osmotic stress	Skriver and Mundy (1990)
The molecular basis of dehydration tolerance in plants	Ingram and Bartels (1996)
The discovery, scope and puzzle of desiccation tolerance in plants	Alpert (2000)
Desiccation tolerance in the resurrection plant *Craterostigma plantagineum.* A contribution to the study of drought tolerance at the molecular level	Bartels and Salamini (2001)
Mechanisms of plant desiccation tolerance	Hoekstra et al. (2001)
Desiccation tolerance studied in the resurrection plant *Craterostigma plantagineum*	Bartels (2005)
ABA signalling in stress-response and seed development	Nakashima and Yamaguchi-Shinozaki (2013)
Surviving metabolic arrest: photosynthesis during desiccation and rehydration in resurrection plants	Challabathula et al. (2016)
Angiosperm plant desiccation tolerance: hints from transcriptomics and genome sequencing	Giarola et al. (2017)
Octulose: a forgotten metabolite?	Zhang and Bartels (2017)
The dynamic responses of cell walls in resurrection plants during dehydration and rehydration	Chen et al. (2020)
Desiccation tolerance: avoiding cellular damage during drying and rehydration	Oliver et al. (2020)
Systems biology of resurrection plants	Gechev et al. (2021)

^a^We apologise to all the authors whose reviews could not be cited due to space limitations

## Why we chose to work with *C. plantagineum*

Until 1990, resurrection plants were not known to a broad scientific audience, and research on them had not received much attention. The description of the characteristics of resurrection plants at a botanical congress in Australia caught our attention, and their exceptional features prompted us to focus our research on these plants. We tried to obtain resurrection plants from botanical gardens or other collections, but realized that it was not easy to obtain sufficient material for molecular experiments. Therefore, we began a long-term collaboration with Prof. E. Fischer (University of Koblenz, Germany), who specializes in systematic studies of African plants. He was able to provide us with specimens of desiccation-tolerant plants from different habitats. His deep knowledge of the African flora also made it possible to get access to close relatives of *C. plantagineum*, which were desiccation sensitive and could later be used for comparative studies (VanBuren et al. 2018). We soon realized that each resurrection plant species requires specific cultivation conditions. *C. plantagineum,* which we originally obtained from Prof. Volk (University of Würzburg, Germany), was a species that we could grow from seeds and propagate under controlled environmental conditions. Interestingly, we noted that not only plantlets emerged from seeds sown in vitro, but also that callus developed (Bartels et al. 1990; Bartels 2005). We examined whether undifferentiated callus similarly to whole plants could be used to study desiccation tolerance. Callus per se did not withstand rapid desiccation, but pretreating it with ABA induced desiccation tolerance (Fig. 1b), which was accompanied by major transcriptional changes. Therefore, plants of *C. plantagineum* in combination with callus appeared to be ideal to study the molecular basis of desiccation tolerance by representing two different systems from the same species (Bartels et al. 1990). The importance of *C. plantagineum* as a model plant for studying desiccation tolerance since 1990 is evidenced by the greatest number of publications for it compared with other model systems (Tebele et al. 2021).

## Molecular analysis of desiccation tolerance in *C. plantagineum*

The article of Bartels et al. (1990) was the first to specifically focus on the molecular identification of transcripts and gene products that are induced by drying in the resurrection plant *C. plantagineum*. The study used, in parallel, detached leaves that were air-dried and ABA-treated, dried callus (Fig. 1). This approach allowed the separation of desiccation-related and non-desiccation-related transcripts to be compared in two metabolically different tissues that both could withstand desiccation.

A differential screening approach compared mRNA from untreated leaves or air-dried leaves and from untreated callus and callus cultured on ABA and then dried. This resulted in the identification of cDNA clones that were only expressed in desiccation-tolerant tissues, and these were grouped into different families (Bartels et al. 1990). The expression of several of the most abundantly represented clones was tested, and those were selected for further analysis that corresponded to transcripts that were rapidly induced in dehydrated tissues and whose expression decreased following rehydration. The desiccation-related mRNAs were induced to different levels and with varying induction kinetics. Representative cDNA clones from different groups were sequenced (Piatkowski et al. 1990) and the proteins they encoded could be grouped into protein categories, with known and unknown functions. Over the years, the number of unknown proteins became fewer as functions could be assigned to them. One main insight from the early characterization of the desiccation-related transcripts was that many encoded proteins were homologous to proteins that abundantly accumulated during seed development. The largest group was that of late embryogenesis abundant (LEA) proteins (Ingram and Bartels 1996). This suggested similarities in desiccation tolerance mechanisms between seeds and vegetative tissues of resurrection plants. Most transcripts and proteins could not only be induced by dehydration, but also by ABA, which confirmed that the ABA response pathway is also a main component of desiccation tolerance in vegetative tissues of resurrection plants and seeds.

By revealing genes involved in desiccation tolerance, such as *Lea* genes, the 1990 study of *C. plantagineum* paved the way for isolating the respective promoter sequences and studying their *cis*-regulatory activation mechanisms, which was the starting point for constructing gene regulatory networks involved in desiccation tolerance. The regulatory mechanisms revealed that desiccation tolerance involves the induction and suppression of a complex set of genes and transcription factors that control groups of genes with similar expression kinetics in response to desiccation (Oliver et al. 2020; Gechev et al. 2021).

It was not possible to obtain knockout mutants with a phenotype by mutagenesis of *C. plantagineum* due to its octoploid genome. Therefore, the best experimental strategy for a functional genetics approach was to generate dominant mutations that lead to a phenotype. Here, the callus system offered the advantage that it could be transformed with *Agrobacterium*, and this allowed T-DNA activation tagging to obtain dominant mutants. This led to the identification of transgenic callus that was desiccation tolerant in the absence of prior ABA treatment and that constitutively expressed *Lea* genes (Furini et al. 1997). The gene whose overexpression was responsible for this phenotype, *Craterostigma* desiccation tolerant-1 (*CDT-1*), could potentially encode a 22-amino-acid polypeptide, but further genomic analyses and the inability to detect a functional CDT-1 protein suggested that it functions as a regulatory non-coding RNA. This revealed early on that not only the classical *trans*-activation of regulatory genes is involved in conferring desiccation tolerance, but also regulation by retro-transposon-like non-coding RNAs. These elements might be species-specific sequences, because no close homologues have been identified in the genomes of other desiccation-tolerant plants.

In contrast to non-coding RNAs, genome analyses during the last 10 years demonstrated that high copy numbers of *Elip* (early light-inducible protein) genes are a constitutive component of vegetative desiccation tolerance in homoiochlorophyllous resurrection plants*,* which retain chlorophyll during desiccation (VanBuren et al. 2019). In *C. plantagineum*, about 200 genes encoding ELIP proteins are present in large clusters throughout its genome (VanBuren et al. 2023) and high copy numbers of *Elip* genes have evolved through tandem duplications and genome duplication. The *Elip* genes are highly expressed upon dehydration in vegetative tissues, and their encoded proteins presumably protect tissues from oxidative stress, which is exacerbated by drying.

On the basis of the examples described above, we would like to suggest that the evolution of vegetative desiccation tolerance has been due to two main factors: first, the amplification and abundant expression of genes derived from existing pathways such as seed maturation (*Lea* genes) or *Elip* genes from light-regulated pathways; second, recently evolved, species-specific regulatory small RNAs such as CDT-1.

## Vegetative desiccation tolerance shares important parameters with seed desiccation tolerance

Until late 1980, desiccation tolerance had been mainly studied in seeds, which acquire desiccation tolerance at a specific developmental stage (Bewley et al. 1989). Several groups characterized ABA-responsive genes that accumulated during desiccation in developing seeds. The majority of the proteins have characteristic biochemical features: they are very hydrophilic, they often lack cysteine or tryptophan residues but do not show homologies to known proteins, and therefore, they were termed LEA proteins according to their expression patterns (Ingram and Bartels 1996; Gechev et al. 2021). LEA proteins are a heterogeneous group of proteins and can be divided into several classes (Hundertmark and Hincha 2008; Gechev et al. 2021). Several genes encoding LEA proteins were identical to so-called *responsive to ABA* (*rab*) genes that were discovered by differential screening of tissue treated with ABA and were not only present in developing seeds, but were also induced by osmotic stress in other tissues of different species (reviewed by Skriver and Mundy 1990). Overexpression of *Lea* genes in planta and in vitro experiments supported that some, but possibly not all LEA proteins have a protective function during water deficit or salt stress (Amara et al. 2013; Banerjee and Roychoudhury 2016; Gechev et al. 2021). One inherent complication of using developing seeds as a model system for studying desiccation tolerance is that it is difficult to distinguish the acquisition of desiccation tolerance from the effects of other concomitant developmental pathways. Therefore, the utilization of desiccation-tolerant callus was an important breakthrough as a research approach (Fig. 1b).

The molecular analysis of desiccation tolerance in vegetative tissues of *C. plantagineum* revealed the synthesis of many components that are shared with desiccation tolerance in seeds (Giarola et al. 2017). Desiccation tolerance in seeds is strictly developmentally regulated such that it is acquired at a precise stage during seed maturation and is abruptly lost at the onset of germination. The balance of the two phytohormones ABA and GA is an important parameter that leads to the induction and subsequent loss of desiccation tolerance in seeds. Both phytohormones regulate the acquisition and loss of desiccation tolerance via triggering specific genetic networks (Sajeev et al. 2024).

The occurrence of common components in vegetative desiccation tolerance in vegetative tissues of resurrection plants and seeds was subsequently supported by studies by several research groups (Farrant and Moore 2011). This led to the current hypothesis that the genetic information for desiccation tolerance is present in the genome of most plants, but that the expression of the corresponding genes in vegetative tissues is restricted to the small group of resurrection plants under natural conditions. One possible reason for this might be that desiccation tolerance is correlated with disadvantages such as slow growth and low biomass. It has also been observed that this extreme adaptation leads to genome duplications and tandemly duplicated selected genes in many resurrection plants, which might have presented a selective disadvantage during evolution.

## *C. plantagineum* and other resurrection plants are sources of metabolites with biotechnological potential

As well as molecular and genetic studies, biochemical analyses were initiated to identify the specific features of *C. plantagineum* and other resurrection plants. These studies revealed that in addition to the induction of specific proteins during desiccation, major metabolic changes occur, which include primary and secondary metabolites. Major pathways such as carbohydrates, lipids or cell wall composition are modulated during desiccation (Bianchi et al. 1991; Gasulla et al. 2013; Jung et al. 2019). An example of the primary metabolites is the accumulation of sucrose in most resurrection plants. *C. plantagineum* has a distinct carbohydrate metabolism that is modulated during desiccation and rehydration (Bianchi et al. 1991; 1992). Fully hydrated leaves, but not roots, contain very high levels of the unusual C8 sugar octulose, which is metabolized to sucrose during dehydration, and during rehydration the opposite occurs and the octulose level increases and that of sucrose decreases (Bianchi et al. 1991). Octulose does not interfere with primary carbohydrate metabolism, and thus the rapid synthesis of sucrose upon water loss does not have negative effects on metabolism. Accumulation of high concentrations of sucrose or trehalose during desiccation is common across different organisms from microorganisms to plants (Bartels and Hussain 2011; Kaiser et al. 1985). Both carbohydrates contribute to desiccation tolerance, because they prevent protein denaturation and membrane fusions (Crowe et al. 1984, 1998). Interestingly, some carbohydrates might have a signalling role in the context of desiccation tolerance. Trehalose was demonstrated to trigger autophagy to prevent cell death during desiccation in the resurrection grass *Tripogon loliiformis* (Williams et al. 2015). In addition, exogenous application of lactose improved the rapid induction of desiccation tolerance in the resurrection plant *Boea hygrometrica*, with an effect on ABA signalling and autophagy (Sun et al. 2025).

A diverse spectrum of metabolites that accumulate during desiccation has been identified in different resurrection plants. Some of these may serve as osmoprotectants, whereas others might partially replace water molecules due to the presence of hydroxyl groups, interact with membranes, or scavenge reactive oxygen molecules. Passon et al. (2020) recently reported that the accumulation of the polyphenol verbascoside, which may act as an antioxidant, correlates with the degree of desiccation tolerance in *C. plantagineum* and its close relatives.

Some of the metabolites may have a potential biotechnological application. For example, the resurrection plant *Myrothamnus flabellifolia* accumulates the polyphenol 3,4,5-tri-O-galloylquinic acid (Bianchi et al. 1993) which inhibits HIV and MLV reverse transcriptases (Gechev et al. 2021). Another example is *Haberlea rhodopensis*, which synthesizes a myconoside that improves skin regeneration and can be applied in cosmetic products (Dell’Acqua and Schweikert 2012; Gechev et al. 2021). These examples show that much potential exists to exploit the secondary metabolites of resurrection plants.

## Future desiccation research perspectives

Much progress over the last 35 years has been made in describing the physiological and molecular genetic characteristics of desiccation-tolerant species across disparate phylogenetic clades. Studies have identified core transcripts, proteins and metabolites that are conserved among diverse desiccation-tolerant species. However, differences are also present among species, which leaves many opportunities to explore the intricate and nuanced mechanisms of desiccation tolerance exhibited by resurrection plants. Orthodox seeds and angiosperm resurrection plants share many components to deal with extreme water loss, but questions remain concerning how resurrection plants activate seed-associated desiccation tolerance mechanisms in vegetative tissues, and which master regulators orchestrate these mechanisms.

Areas of vegetative desiccation tolerance that could be further researched include natural intraspecific variation across populations and variation across tissues and life stages of an individual plant. In addition, the role played by chromatin organization and epigenetic mechanisms in modulating desiccation tolerance remains underresearched. Future research should prioritize the importance of interdisciplinary systems biology approaches, to elucidate the complex interplay among the genome, transcriptome, proteome, metabolome and consequent physiological output in desiccation tolerance.

Publicly available whole-genome assemblies exist for several resurrection plants (Gechev et al. 2021), and comparative genomic studies will help to identify common genome signatures related to desiccation tolerance. A notable recent metagenomics approach has created the “Drying without Dying” plant genome database containing the genomes of six desiccation-tolerant species and ten closely related genomes (Gao et al. 2024), which will facilitate the comparison of desiccation tolerance at the whole-genome level. Moreover, more desiccation-tolerant species are being identified (e.g. Porembski 2021; Smrithy et al. 2023), which will potentially provide more resources for future studies.

One of the most topical and relevant goals is how traits of resurrection plants can be used to genetically re-programme crop species while minimizing detrimental effects on growth or yield. Transgenic approaches to engineer crop species for improved drought tolerance can either introduce genes known to encode important components of the response, or activate the inherent seed drought tolerance that is suppressed in vegetative tissues. Many approaches have attempted to introduce genes into crop species, or to overexpress regulators or protective molecules, with some limited success in improving their ability to survive water loss (reviewed in Umezawa et al. 2006). However, due to the complex basis of desiccation tolerance, it is likely that a combination of classical breeding methods and biotechnological approaches is required to produce crops that better survive periods of drought (Raza et al. 2023). In this context, the potential of gene editing to simultaneously modulate the expression of suites of genes in a species in a targeted way is promising and may also find more public acceptance than transgenic approaches (reviewed in Sami et al. 2021). Gene-editing technologies applied to resurrection plants will most likely further advance our understanding of desiccation tolerance because many resurrection plants have large genomes and high ploidy, which hampers classical reverse-genetic approaches. Gene editing could accelerate functional genomics, if the ability to transform resurrection species is optimized. Applying genomic discoveries and functional insights from resurrection species to engineer crop plants will potentially improve global food security in the context of climate change.

## Data Availability

This review article does not include any new data.

## Abbreviations

CDT-1: *Craterostigma* desiccation tolerant-1
DS: Desiccation sensitive
DT: Desiccation tolerant
Elip: Early light-inducible proteins
Lea: Late embryogenesis abundant
